# Re-expansion pulmonary edema after resection of cerebellar lesion in a patient with bronchial occupying lesion: A case report

**DOI:** 10.1097/MD.0000000000015046

**Published:** 2019-04-12

**Authors:** Jiang Yu, Ying Wang, Xuanyu Chen, Ruozhu Cheng, Xueli Yang, Hongzhi Chen

**Affiliations:** Department of Anesthesiology, Shengjing Hospital Affiliated to China Medical University, Shenyang, China.

**Keywords:** bronchial occupying lesion, bronchospasm, case report, cerebellar lesions, re-expansion pulmonary edema

## Abstract

**Rationale:**

Re-expansion pulmonary edema (RPE) is a non-cardiogenic pulmonary edema, and is secondary to pulmonary collapse caused due to various reasons. However, RPE is rarely encountered during non-thoracic surgeries and is associated with much higher risk than that occurring in thoracic surgeries.

**Patient concerns:**

Herein we have reported a case report of a 55-years-old male patient. Preoperative examination indicated occupying lesions in the bronchus and cerebellar hemisphere. Under general anesthesia, the patient received resection of cerebellar lesion and developed acute atelectasis, and RPE occurred when cannulation was withdrawn after re-expansion. Supportive and symptomatic treatment was given to the patient for recovery well.

**Diagnosis:**

RPE.

**Interventions:**

The trachea was cannulated and connected to a ventilator for assisted ventilation. The patient was also given symptomatic treatment including nebulization, diuresis, and anti-inflammation.

**Outcomes:**

The patient recovered well and was discharged on day 8 after surgery.

**Lessons:**

Patients with occupying lesions of the airway should undergo bronchoscopy to determine the location, size, and distance of the lesion from the incisors. The anesthesiologists should determine appropriate anesthetic regimens according to the examination results to avoid acute atelectasis and postoperative pulmonary edema.

## 1 Introduction

Re-expansion pulmonary edema (RPE) refers to pulmonary edema when the lung tissue re-expands after collapse. The onset of RPE is acute, and the patients might experience a sharp declination in oxygen saturation, hypotension and shock might be seen in some seriously affected patients, threatening their life.^[1]^ Previous studies have reported RPE after thoracic surgery, thoracentesis, or pneumothorax,^[2,3]^ but is rarely seen in other occasions. In this study, we reported a case that had bronchial occupying lesions before surgery and developed RPE due to bronchospasm-induced transient atelectasis. The informed consent was obtained from the patient for publication of this manuscript.

## 2 Case report

**Figure 1:**
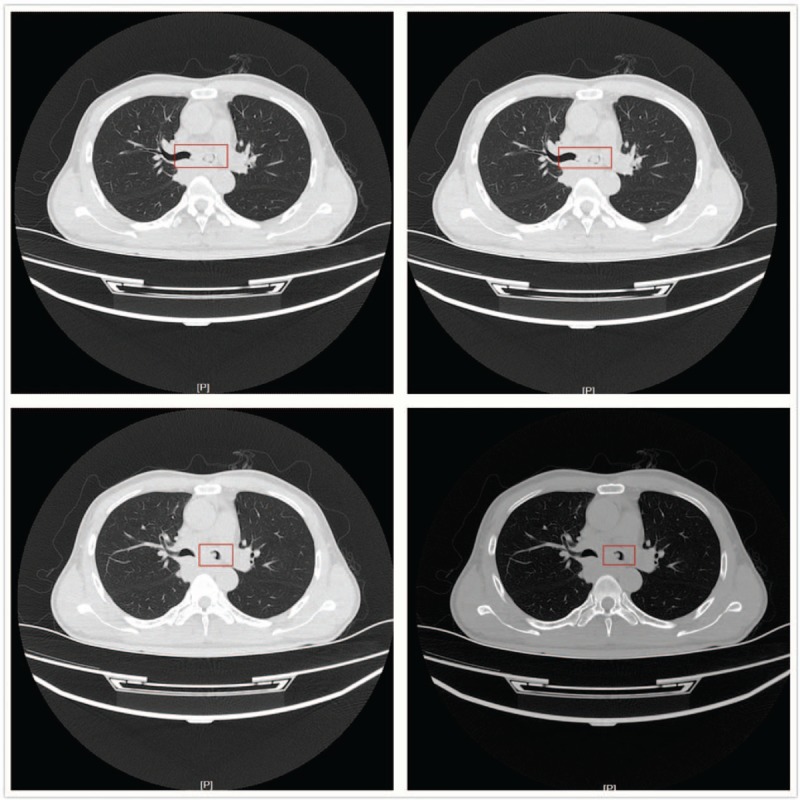
Chest CT before operation. CT = computed tomography.

We reported a case report of a 55-years-old male who received resection of right cerebellar occupying lesions 3 years ago. Postoperative pathology suggested large cell neuroendocrine carcinoma of the lung. In the past year, the patient had instable gait, dizziness, headache, and cough, and visited our hospital due to continuously aggravating symptoms. Positron emission tomography-computed tomography (PET-CT) examination suggested left cerebellar lesion with local hemorrhage, and chest computed tomography (CT) suggested soft tissue nodules in the left and right main bronchi (Fig. 1). Based on relevant examinations, the patient was diagnosed to have recurrence of pulmonary large cell neuroendocrine carcinoma with intracranial metastasis. As the patient had a special lesion in the airway, our anesthesiologist advised the patient for further detailed examination. Yet during the preparation of examination, the patient developed aggravated conditions of sleepiness and dyspnea, and blood gas analysis indicated type II respiratory failure. Thus, emergency operation was performed immediately.

**Figure 2:**
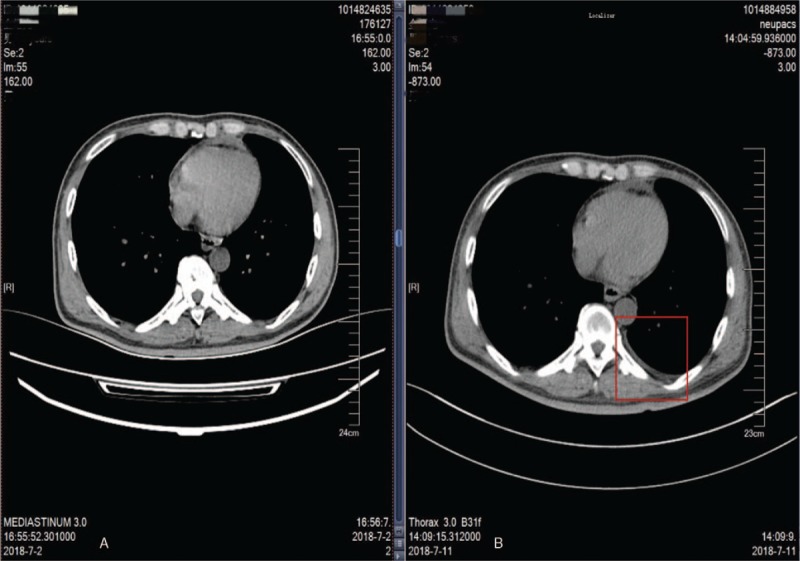
Chest CT of different periods. (A) Chest CT before RPE. (B) Chest CT after RPE. CT = computed tomography, RPE = re-expansion pulmonary edema.

After entering the operation room, the patient was given intravenous drip of compound lactated Ringer's injection at 7 mL/kg/h. EGF monitoring and dorsalis pedis artery cannulation were performed for monitoring SpO_2_ and arterial pressure and provided a mask for oxygen inhalation at 6L/min. The baseline characteristics of the patient showed BP of 145/85 mmHg, SpO_2_ 94%, HR 110 beats/min, and ECG showed no abnormalities. Anesthesia induction was performed using intravenous administration of sufentanil 20 μg, etomidate 20 mg, and rocuronium 50 mg. After 60 seconds of assisted ventilation with low tidal volume, a #7.5 tube was cannulated under glidescope guidance, and the depth was fixed at 23 cm. Auscultation of both lungs showed no significant abnormalities, so the ventilator was connected, the mode was set as mechanical ventilation, VT at 400 mL, and RR at 13 times/min. Anesthesia was maintained by 1.5%∼2.5% sevoflurane in combination with 50% O_2_ and 50% N_2_O, and intravenous infusion of remifentanil was also given to maintain BP within 120 to 140 mmHg, HR 80 to 100 beats/min, SpO_2_ 95% to 100% and PETCO_2_ 25 to 30 mmHg. Intraoperative hemodynamics remained stable, and the arterial blood gas analysis showed no significant abnormalities. However, during the operation, the patient showed 3 times of unexplained SpO_2_ declination, and the lowest level reached to 90%. After giving lung expansion, the SpO_2_ was increased to 95%. During surgery, the patient was asked to lie in a prone position, and the operation lasted for 3 hours and 47 minutes. 1.7U of red blood cells was infused, and the volume of compound lactated Ringer's injection was 1260 mL, colloid 1000 mL, and urine 450 mL. When the patient was recovered from anesthesia and showed spontaneous breathing, 1 mg of neostigmine was used for antagonizing muscle relaxation, and 0.5 g of doxapram for the excitation of respiratory center. However, when performing sputum suction, the sucking-tube was obstructed, and the SpO_2_ was dropped rapidly to 80% after sputum suction. Immediately, lung expansion with pure oxygen was performed, which led to no remission, but the SpO_2_ further declined to 55%. We then auscultated both the lungs and found no breathing sounds in the hilum, upper and lower lobes of the left lung. Blood gas analysis suggested pH: 7.281, PaCO_2_: 37.4mmHg, PO_2_: 38.8 mmHg, and Lac:4.2mmol/L. Thus, an intravenous infusion of 120 mL sodium bicarbonate was given, and lung expansion was continued with 100% O_2_ as well as sevoflurane. About 8 minutes later, the SpO_2_ was increased gradually to 100%. After that, the patient was sent to ICU, and he showed intolerance to tracheal cannulation approximately 20 minutes later. The doctor even after withdrawing the cannulation, the patient again showed breathing difficulty and sudden drop of SpO_2_, with the lowest level of 65%, and oxygen mask failed to relieve the condition. Auscultation of both lungs at the moment showed moist rales of the left lung, and the patient had much white foam secretions from his mouth. After full oral cavity aspiration, the trachea was cannulated and connected to a ventilator for assisted ventilation. The patient was also given symptomatic treatment including nebulization, diuresis and anti-inflammation, and reached weaning indications after 3 days of operation. Auscultation suggested weak respiratory sounds in the hilum and upper field of the left lung, and some moist rales were still audible from the lower left lung. The ventilator was weaned and cannulation was withdrawn, and the patient underwent CT examination. The results showed no abnormalities except for a slight increase in the amount of fluid in the left thoracic cavity (Fig. 2). After that, the patient showed no breathing difficulties and was discharged on day 8 after surgery.

## 3 Discussion

The mechanism of RPE is similar to that of acute respiratory distress syndrome (ARDS). To be specific, when the lung is expanded, mechanical damages would increase the permeability of pulmonary capillaries and uplift the hydrostatic pressure. This, in turn, causes outflow of fluid and protein from the pulmonary vessels to the interstitial lung, resulting in pulmonary edema.^[4]^ The development of RPE is non-specific and is mostly unilateral, but at times involves both lungs. RPE often occurs during or after several hours of lung expansion.^[5]^ But a study reported positive correlation between the incidence of RPE and the degree as well as time of lung collapse.^[6]^ After RPE, the patients often reported severe cough, chest tightness, breathing difficulty, and white foam sputum; moist rales of the affected side heard on auscultation, and severe cases might even have hemodynamic disorders and death.^[7]^

Currently, there is no special method for RPE diagnosis and is determined mainly by glass-like changes of diseased vessels on pulmonary CT images. However, for acute RPE, immediate respiratory support should be provided. CT scanning cannot be performed immediately, as the conditions are stabilized and the special imaging features would disappear, leaving only a slight amount of pleural effusion.

**Figure 3:**
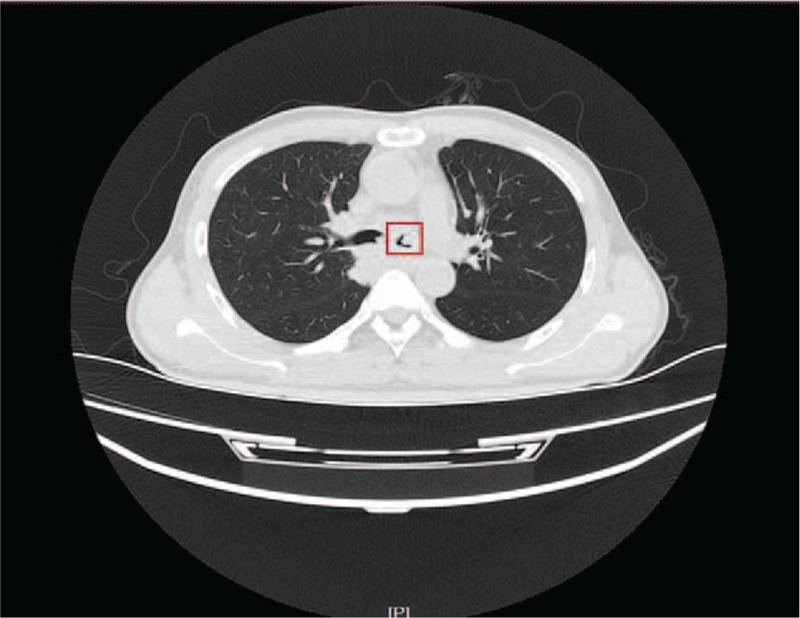
Chest CT on left main bronchus level before operation. CT = computed tomography.

**Figure 4:**
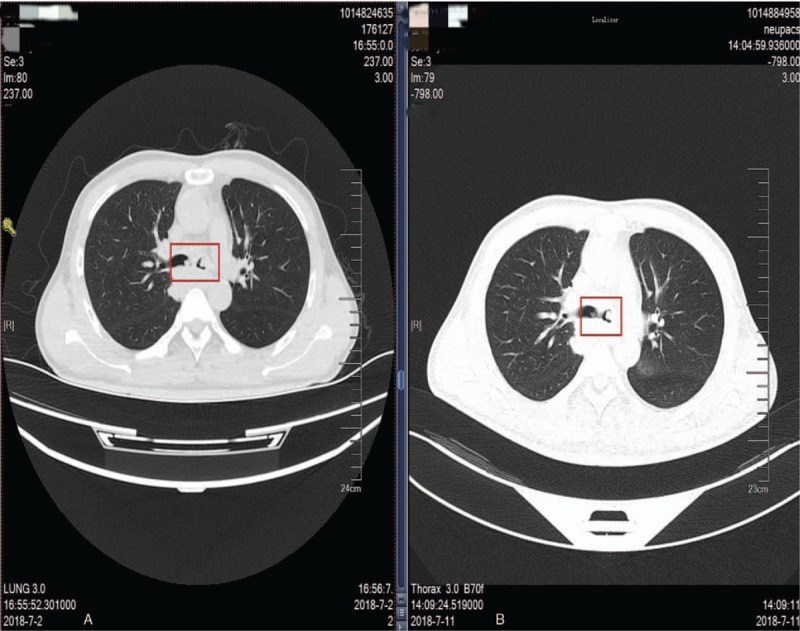
Chest CT at left main bronchus level of different periods. (A) Chest CT at left main bronchus level before RPE. (B) Chest CT at left main bronchus level after RPE. CT = computed tomography, RPE = re-expansion pulmonary edema.

In the reported case, dyspnea and white foam sputum occurred 1 hour after atelectasis. This in combination with later imaging features (pleural effusion), we speculated that the patient had RPE. Yet it was different when compared with the previously reported cases, as this patient did not receive any chest surgery or operation, and had a history of atelectasis before surgery. Therefore, his RPE occurred in ICU due to transient atelectasis during the recovery period from anesthesia, and the reason why the patient showed atelectasis is the focus of our discussion. Reviewing the whole process, we found that the obstruction of the left bronchus was the direct cause of atelectasis during the recovery period. There are 2 main reasons for airway obstruction, 1 due to the blocks of the same diameter as the airway, and the other include airway contractures due to various reasons. A closer look at the CT images before operation showed an occupying lesion of large diameter in the left main bronchus, which occupied 3/4 of the diameter of the whole airway (Fig. 3). If the lesion was decreased during the recovery period, it probably would cause bronchial obstruction. However, postoperative CT examination suggested that the lesion remained still, and demonstrated no changes in its morphology or location (Fig. 4). Therefore, the patient had reduced airway diameter due to bronchospasm, and because of the presence of occupying lesion, obstruction of the left main bronchus finally led to atelectasis of the left lung.

Bronchospasm is a common perioperative complication. Its main manifestations include contraction of the bronchial smooth muscle and increased resistance of the airway, and occlusion of the whole airway may occur in severe cases.^[8]^ The mechanisms of bronchospasm involve airway hyper-responsiveness and inflammatory reactions.^[9]^ In this case, the related factors causing bronchospasm are:

1) the patient had a coughing history for years due to bronchial occupational exposures, causing inflammation of the airway;
2) sputum suction under light anesthesia was a common reason for bronchospasm.

In this case, sputum suction might cause vagal excitation, leading to intense contraction of the smooth muscles. Fortunately, our anesthesiologist performed timely and effective treatment for the patient, and so the airway spasm was quickly relieved, and the time of atelectasis was reduced.

At present, the main treatment for RPE is to maintain adequate oxygen supply. Non-invasive nasal catheter and oxygen mask can be used to improve hypoxemia, and invasive airway cannulation or ventilator therapy can be performed as soon as possible. In addition to oxygen supply, glucocorticoids should be given for anti-inflammatory treatment.^[10]^ Under stable hemodynamics, a negative fluid balance should be maintained to reduce pulmonary edema.^[11]^ Since RPE was diagnosed and treated immediately, the patient was recovered well in the following days and showed no other complications.

## 4 Conclusion

For patients with airway occupying lesions and those planning for general anesthesia, the size and location of the lesions should be determined before operation to develop appropriate anesthetic regimens. This could avoid the fall off of the occupying lesions, and also reduce the airway stimulation during the perioperative period to prevent spasm. If atelectasis occurs for any reason, we should be vigilant for the development of RPE after pulmonary re-expansion.

## Author contributions

**Data curation:** Ying Wang.

**Project administration:** Ruozhu Cheng.

**Resources:** Hongzhi Chen.

**Software:** Xuanyu Chen.

**Writing – review & editing:** Jiang Yu, Xueli Yang.
